# Genetic Targeting of Arginase-II in Mouse Prevents Renal Oxidative Stress and Inflammation in Diet-Induced Obesity

**DOI:** 10.3389/fphys.2016.00560

**Published:** 2016-11-22

**Authors:** Ji Huang, Angana Rajapakse, Yuyan Xiong, Jean-Pierre Montani, François Verrey, Xiu-Fen Ming, Zhihong Yang

**Affiliations:** ^1^Cardiovascular and Aging Research, Division of Physiology, Department of Medicine, University of FribourgFribourg, Switzerland; ^2^Swiss National Centre of Competence in Research (NCCR) Kidney Control of Homeostasis “Kidney.CH”Zurich, Switzerland; ^3^Institute of Physiology, University of ZurichZurich, Switzerland

**Keywords:** adhesion molecules, arginase, oxidative stress, obesity, renal disease

## Abstract

Obesity is associated with development and progression of chronic kidney disease (CKD). Recent evidence demonstrates that enhanced levels of the L-arginine:ureahydrolase, including the two isoenzymes arginase-I (Arg-I) and arginase-II (Arg-II) in vascular endothelial cells promote uncoupling of endothelial nitric oxide synthase (eNOS), leading to increased superoxide radical anion and decreased NO production thereby endothelial dysfunction. Arg-II but not Arg-I is abundantly expressed in kidney and the role of Arg-II in CKD is uncertain and controversial. We aimed to investigate the role of Arg-II in renal damage associated with diet-induced obesity mouse model. Wild type (WT) C57BL/6 mice and mice deficient in Arg-II gene (Arg-II^−/−^) were fed with either a normal chow (NC) or a high-fat-diet (HFD) for 14 weeks (starting at the age of 7 weeks) to induce obesity. In WT mice, HFD feeding caused frequent renal lipid accumulation, enhancement of renal reactive oxygen species (ROS) levels which could be attenuated by a NOS inhibitor, suggesting uncoupling of NOS in kidney. HFD feeding also significantly augmented renal Arg-II expression and activity. All the alterations in the kidney under HFD feeding were reduced in Arg-II^−/−^ mice. Moreover, mesangial expansion as analyzed by Periodic Acid Schiff (PAS) staining and renal expression of vascular adhesion molecule-1 (VCAM-1) and intercellular adhesion molecule-1 (ICAM-1) in HFD-fed WT mouse assessed by immunoblotting were reduced in the HFD-fed Arg-II^−/−^ mice, although there was no significant difference in body weight and renal weight/body weight ratio between the WT and Arg-II^−/−^ mice. Thus, Arg-II expression/activity is enhanced in kidney of diet-induced obesity mice. Genetic targeting of Arg-II prevents renal damage associated with obesity, suggesting an important role of Arg-II in obesity-associated renal disease development.

## Introduction

According to the latest Non-Communicable Diseases Risk-Factor Collaboration (NCD-RisC) report, in 2014 there were 422 million people worldwide who had diabetes, i.e., roughly a four-fold increase over the past 35 years (Collaboration, 2016). Diabetic nephropathy or diabetic chronic kidney disease (CKD) is one of the important diabetic complications representing the most common cause of end-stage renal disease (Collins et al., 2013). Although the mechanisms underlying obesity-associated diabetic renal damage are complex and have not yet been fully understood, it has been demonstrated that ectopic renal lipid accumulation, inflammation including macrophage infiltration in kidney through adhesion molecules, i.e., vascular adhesion molecule-1 (VCAM-1) and intercellular adhesion molecule-1 (ICAM-1), glomerulus mesangial expansion, impaired endothelial nitric oxide (NO) bioavailability and oxidative stress or increased ROS generation in kidney are importantly involved in the development of diabetic renal damage (Nakagawa et al., 2011).

There are studies showing key roles of endothelial nitric oxide synthase (eNOS) in protection against development of diabetic renal disease (Wang et al., 2011; Cheng et al., 2012; Cheng and Harris, 2014). Functional alteration of eNOS, in particular, eNOS-uncoupling, a situation that eNOS enzyme produces more superoxide radical anion instead of NO, has been shown to be critical in endothelial dysfunction and diabetic renal disease (Goligorsky et al., 2001). Under eNOS-uncoupling condition, superoxide radical anion reacts with NO, resulting in decreased NO bioavailability and increased generation of the more potent pro-oxidant peroxynitrite causing nitrosative stress (Förstermann and Sessa, 2012). NO is derived from L-arginine and decrease in NO is not only important for pathogenesis of vascular complications but also for progression of renal disease associated with diabetes (Cheng and Harris, 2014). Among other mechanisms, elevated activity and/or expression of arginase i.e., the L-arginine:ureahydrolase including arginase-I (Arg-I) and arginase-II (Arg–II) isoenzyme, has been shown to cause eNOS-uncoupling, resulting in increased oxidative stress in aging and age-associated cardiovascular diseases including atherosclerosis and type 2 diabetic vascular dysfunctions (Yepuri et al., 2012; Li and Förstermann, 2013).

Arg-I and Arg-II are encoded by two separate genes (Sparkes et al., 1986; Gotoh et al., 1997). Arg-I is located in cytosol, while Arg-II is in mitochondrion (Ash, 2004). Despite different subcellular localization, the two isoenzymes share the same substrate L-arginine and compete with eNOS for L-arginine, thereby decreasing intracellular L-arginine bioavailability for production of NO (Yang and Ming, 2013). Under the condition of L-arginine deficiency caused by elevation of either Arg-I or Arg-II, eNOS becomes uncoupled, leading to oxidative stress and endothelial dysfunction in cardiovascular diseases and aging (Li and Förstermann, 2013; Yang and Ming, 2013). Hence, eNOS-uncoupling has been shown to be an important mechanism for oxidative stress in cardiovascular pathology.

It is well known that Arg-I is mainly expressed in hepatocytes and is an essential enzyme involved in hepatic urea cycle for detoxification of ammonia (Crombez and Cederbaum, 2005; Tsang et al., 2012). In contrast, Arg-II is highly expressed in kidney (Yang and Ming, 2014). The function of Arg-II in kidney is, however, less well defined. There is only a limited number of studies indicating that Arg-II is upregulated and is detrimental for renal function in type 1 diabetes animal models (Morris et al., 2011; Toque et al., 2013; You et al., 2013, 2014). However, whether Arg-II is also involved in obesity-associated type 2 diabetic renal disease is not known. In contrast to these studies, a recent study suggests that lack of Arg-II even accelerates some of the diabetes-induced renal damages in type 1 diabetic mouse model (Romero et al., 2013).

The aim of our study is to investigate whether Arg-II is involved in renal damage in a diet-induced obesity mouse model.

## Materials and methods

### Materials

Antibodies against Arg-II (sc-20151), ICAM-1(sc-8439), and VCAM-1 (sc-8304) were purchased from Santa Cruz Technology Inc. (Dallas, USA); Antibody against tubulin (T5168), 3-nitrotyrosine (06-284) and Periodic Acid-Schiff (PAS) staining kit (395B) were from Sigma (Buchs, Switzerland); Antibody against B0AT3 (Slc6a18) was produced and validated as previously described (Romeo et al., 2006). IRDye800-conjugated anti-rabbit IgG (926-32211) was purchased from LI-COR Biosciences (Lincoln, USA); Alexa Fluor 680-conjugated anti-mouse IgG (A21057), Alexa Fluor 488 conjugated-Goat anti-rabbit IgG (H+L) secondary antibody (A-11008) and dihydroethidium (DHE, D23107) were from Invitrogen/Thermo Fisher Scientific (Waltham, MA USA).

### Animal models

Arg-II^−/−^ mice were kindly provided by Dr. William O'Brien (Shi et al., 2001) and backcrossed to C57BL/6J for more than 10 generations (Ming et al., 2012). Genotyping was performed by polymerase chain reaction (PCR) as previously described (Shi et al., 2001). The WT and Arg-II^−/−^ offsprings from hetero/hetero cross were interbred to obtain WT and Arg-II^−/−^ mice, respectively, for experiments. Starting at the age of 7 weeks, the male WT and Arg-II^−/−^ mice were given free access during 14 weeks to either a normal chow (NC; energy content: 10.6% fat, 27.6% protein, and 57% carbohydrate, fiber 4.8%; Provimi Kliba NAFAG 3436; Kaiseraugst, Switzerland) or a high fat diet (HFD, energy content: 55% fat, 21% protein, and 24% carbohydrate; Harlan Teklad TD 93075; Horst, Netherlands). Animals were sacrificed after 14 weeks of HFD feeding. The kidneys were collected for histological analysis or snap-frozen in liquid nitrogen and kept at −80°C until used for immunoblotting analysis and arginase activity assay or cryosectioning. Due to the fact that not all the experiments can be done with the same kidney samples for different purposes such as immunoblotting, enzymatic activity assay, oxidative/nitrosative stress measurement, and immunofluorescence staining, PAS staining, etc., various series of experiments with mice on NC and HFD were initiated. The protocol of animal handling and experimentation was approved by the Service de la sécurité alimentaire et des affaires vétérinaires, Etat de Fribourg, Switzerland.

### Oil Red O staining

Cryosections of kidneys (7 μm thick) were fixed with 4% paraformaldehyde for 10 minutes followed by briefly washing with running tap water. After quickly rinsing with 60% isopropanol, the sections were stained with freshly prepared and filtered Oil Red O working solution for 15 minutes, followed by rinsing with 60% isopropanol and washing with distilled water (Ramírez-Zacarías et al., 1992). Images were obtained with Zeiss microscope.

### Mesangial expansion

3-μm paraffin sections of kidneys were stained with periodic acid Schiff (PAS) staining kit according to manufacturer's instruction. All images were obtained with Zeiss microscope. The mesangial expansion was analyzed by percentage of PAS-positive material which occupies the mesangial matrix within the tuft. A minimum of 20 glomeruli in each specimen was examined.

### Kidney arginase activity assay

Arginase activity in kidney lysates was measured by colorimetric determination of urea formed from L-arginine in an *in vitro* activity assay as previously described (Xiong et al., 2013). Briefly, kidney powders were lysed in lysis buffer containing 10 mmol/L Tris-HCl (pH 7.4), 0.4% Triton X-100, 10 μg/mL leupeptin, and 0.1 mmol/L phenylmethylsulfonyl fluoride (PMSF). Samples were centrifuged at 16,200 × g at 4°C for 10 min, and protein concentration of the supernatant was determined by the Lowry method (Bio-Rad). For measurement of arginase activity, an equal amount of the cell lysate was added to 50 μL of Tris-HCl (10 mmol/L [pH 7.4]) containing 5 mmol/L MnCl_2_. Arginase was then activated by heating the mixture at 56°C for 10 min. The hydrolysis reaction of L-arginine by arginase was conducted by incubating the mixture containing activated arginase with 100 μL of L-arginine (100 mmol/L [pH 9.6]) at 37°C for 1 h. For colorimetric determination of urea, 1 mL of chromogenic reagent consisting of 1 volume of 3% 2,3-butanedione monoxine and 29 volumes of the acid solution mixture (H_2_SO_4_:H_3_PO_4_:H_2_O 1:3:7) was added, and the mixture was then heated at 100°C for 30 min. After placing the samples in the dark for 10 min at room temperature, the urea concentration was determined spectrophotometrically by the absorbance at 492 nm. The amount of the urea produced was used as an index for arginase activity.

### Immunoblotting

Tissue lysate preparation, SDS-PAGE, and transfer of SDS gels to Immobilon-P membranes (Millipore) were performed as previously described (Ming et al., 2012). The resultant membrane was first incubated with the corresponding primary antibody overnight at 4°C with gentle agitation after blocking with 5% skim milk. After washing, the blot was then further incubated with corresponding anti-mouse (Alexa Fluor 680-conjugated) or anti-rabbit (IRDye 800-conjugated) secondary antibodies. Signals were visualized using the Odyssey Infrared Imaging System (LI-COR Biosciences, USA). Quantification of the signals was performed using NIH Image 1.62 software (US National Institutes of Health).

### Confocal immunofluorescence staining of Arg-II and 3-NT

Kidneys from the WT and Arg-II^−/−^ mice were isolated and fixed with 3.7% paraformaldehyde and embedded in paraffin. After deparaffinization in xylene, hydration in ethanol, and antigen retrieval in Tris-EDTA buffer (10 mmol Tris Base, 1 mM EDTA, 0.05% Tween 20, pH 9.0) in a pressure cooker, paraffin-embedded sections (5 μm) were blocked with 1% BSA in PBS for 60 min and were then incubated with the first antibodies (Arg-II 1:100, B0AT3 1:200, 3-nitrotyrosine 1:200) at 4°C overnight and subsequently with fluorescence-labeled secondary antibodies at room temperature for 2 h, followed by counterstaining with 300 nmol/L DAPI (4′6-diamidino-2-phenyl-indole-dihydrochloride, Invitrogen) for 3 min. The immunofluorescence signals were visualized under LEICA's DIM6000 Confocal microscope. The intensity of the fluorescence was quantified by NIH Image J software and expressed as intensity per arbitrary unit of area.

### Detection of ROS

Detection of ROS levels was performed as described previously (Rajapakse et al., 2011). For measurement of ROS, pre-treated without or with NOS inhibitor L-NAME (1 mmol/L, 1 h), cryosections were incubated with 5 μmol/L DHE in PBS for 30 minutes. Samples were then washed 3 times and images were obtained with Zeiss fluorescence microscope. The intensity of the fluorescence was quantified by NIH Image J software.

### Statistics

Data are given as mean ± SEM. In all experiments, n represents the number of experiments or animals. Statistical analysis was performed with Student's unpaired *t*-test or analysis of variance (ANOVA) with Tukey-test. Differences in mean values were considered significant at two tailed *P* ≤ 0.05.

## Results

### Increased Arg-II expression/activity in HFD-induced obesity

**Figure 1:**
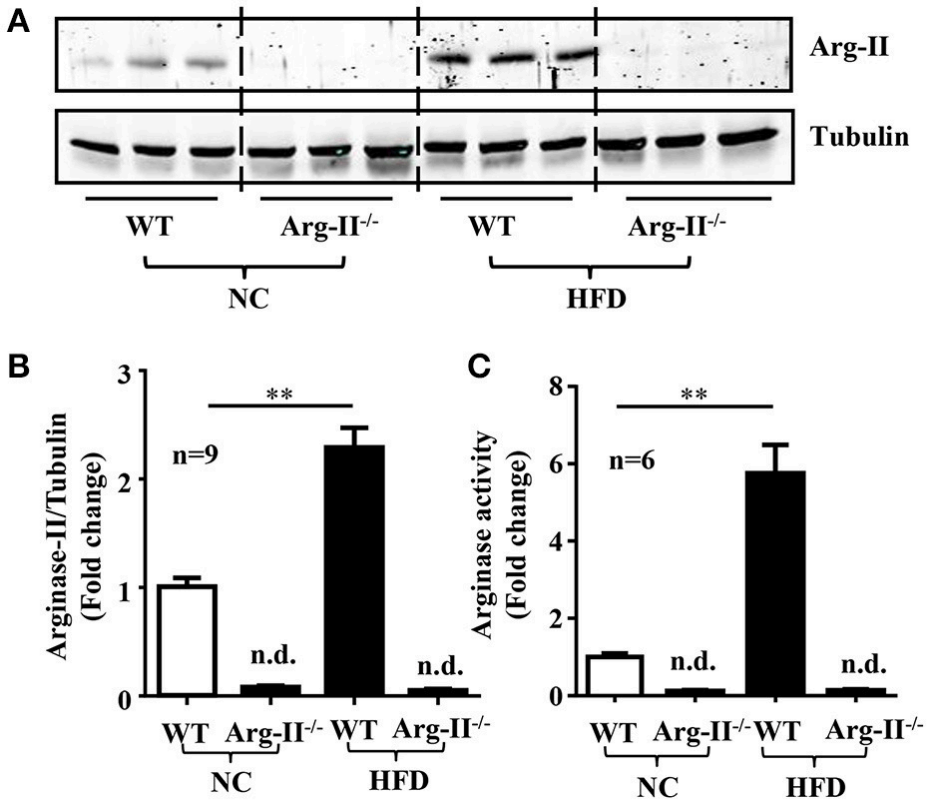
**Enhanced renal Arg-II expression and activity in HFD-induced obesity. (A)** Immunoblotting analysis of Arg-II expression in WT and Arg-II^−/−^ mice with NC and HFD. **(B)** Bar graphs show quantification of Arg-II protein expression and **(C)** activity. *n* = 9 for protein levels and *n* = 6 for enzymatic activity, ^**^p < 0.01 between indicated groups, n.d., not detectable.

**Figure 2:**
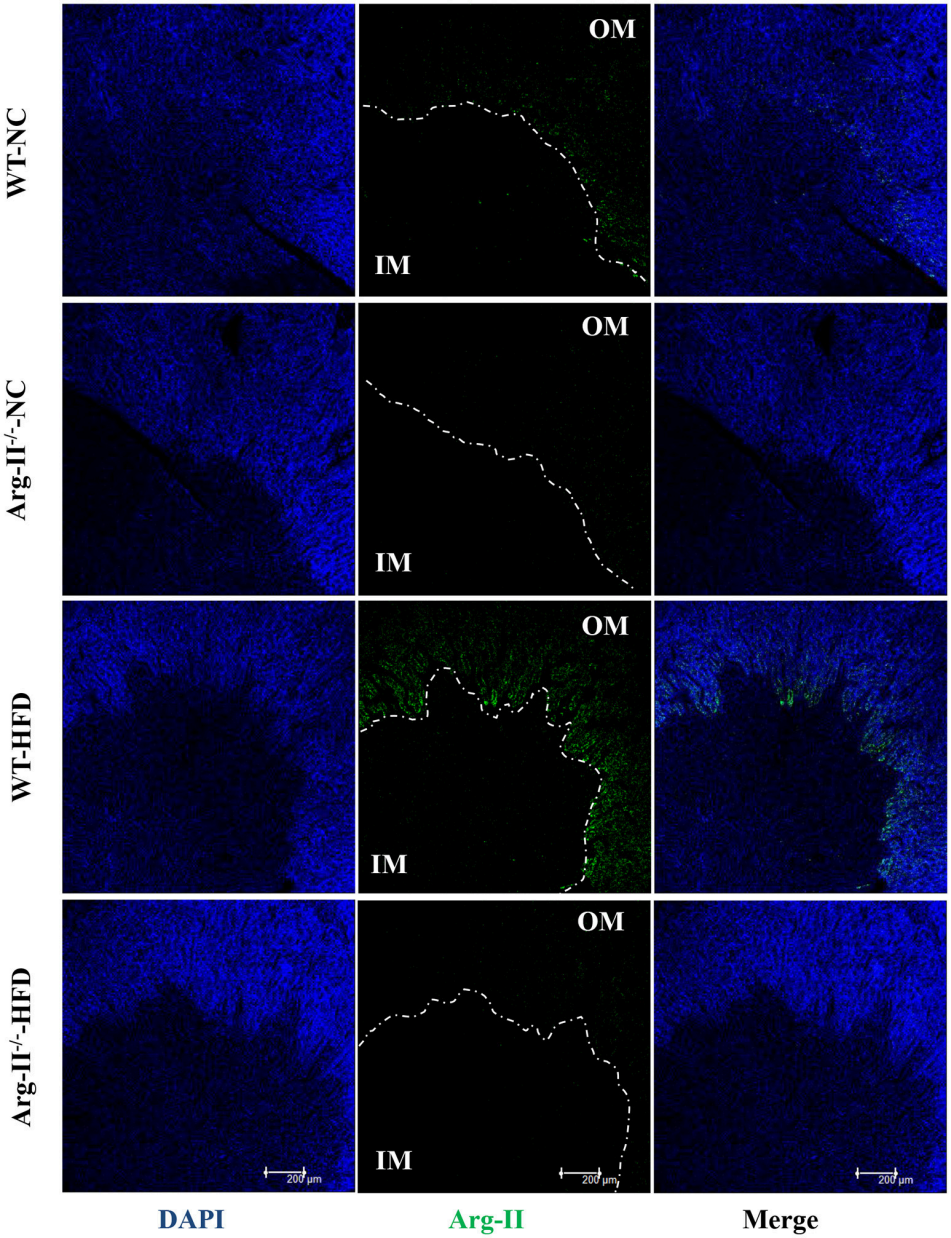
**Arg-II is expressed in outer medulla of kidney**. Confocal immunofluorescence staining of Arg-II (green) and DAPI (blue) in the kidney of WT and Arg-II^−/−^ mice fed either a normal chow (NC) or high fat diet (HFD). Representative images of individual staining and merged images are shown (*n* = 4 for each group). OM, outer medulla; IM, inner medulla.

**Figure 3:**
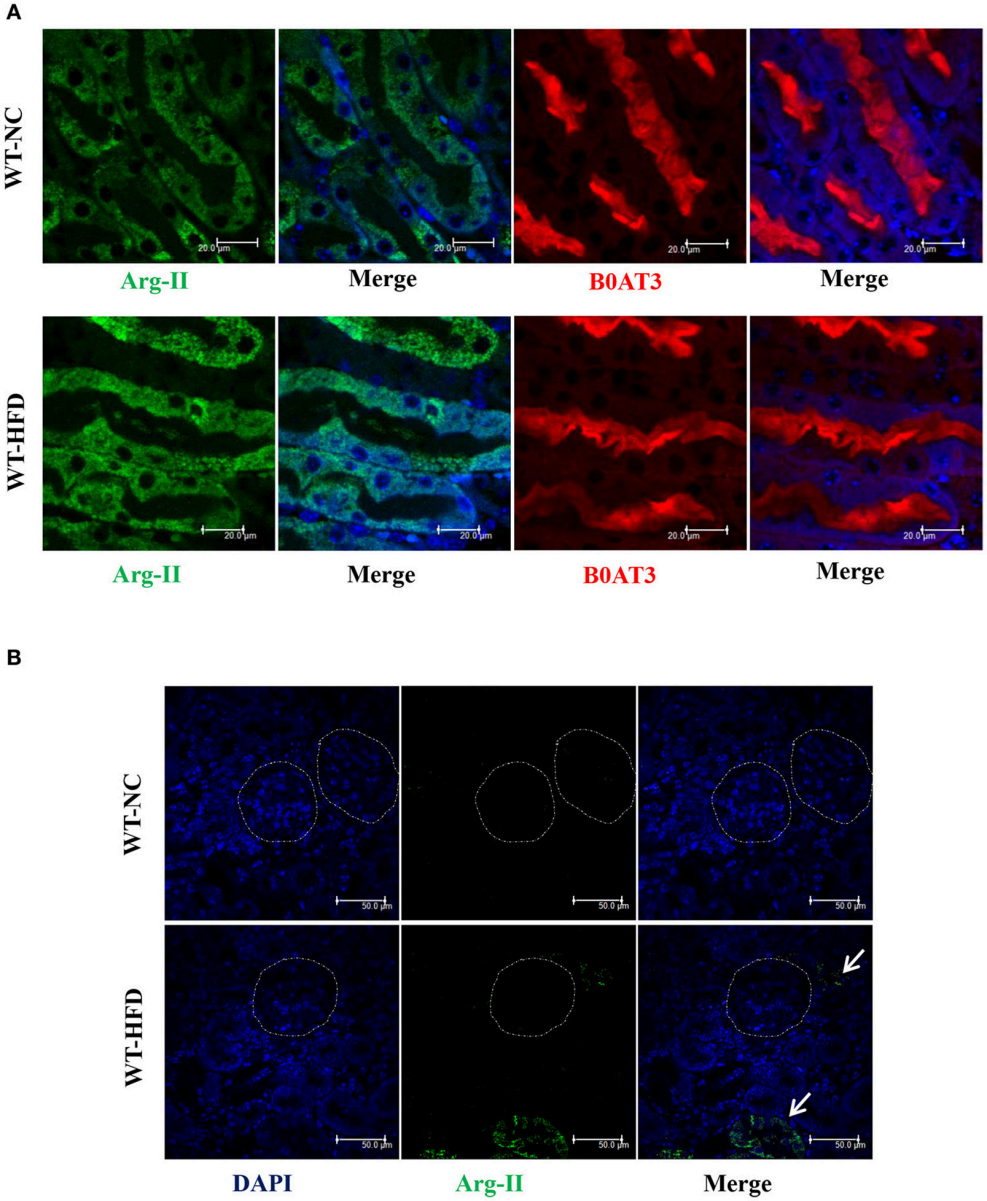
**Arg-II is localized in proximal straight tubules. (A)** Confocal immunofluorescence staining in consecutive sections demonstrates that Arg-II (green) is localized in the S3 proximal straight tubules which are positive for B0AT3 (Slc6a18, red, a luminal marker of S3 proximal straight tubules) in WT mice fed either a normal chow (NC) or high fat diet (HFD). **(B)** No obvious Arg-II (green) signal was detected in glomeruli (circled area) in WT mice fed either NC or HFD. Some Arg-II signals were observed in tubules outside glomeruli (green indicated by arrows). Representative images of individual staining and merged images are shown (*n* = 4).

In the WT mice, HFD feeding significantly enhanced Arg-II expression and arginase activity in kidney (Figure 1). As expected, no Arg-II expression and arginase activity could be detected in Arg-II^−/−^ mouse kidney, demonstrating that renal arginase activity is attributable to Arg-II isoenzyme. Confocal microscopic immunofluorescence staining showed that Arg-II was mainly expressed in tubules of outer medulla, which is increased in the HFD fed WT mice (Figure 2). Further experiments in consecutive sections demonstrated that Arg-II is expressed in the tubule cells which are positive for B0AT3 (Slc6a18), a luminal marker of S3 straight segment of proximal tubules (Figure 3A). No significant signal of Arg-II in the glomeruli could be detected either in mice fed NC or HFD (Figure 3B).

### Arg-II-deficiency protects against ectopic renal lipid accumulation in obesity

**Figure 4:**
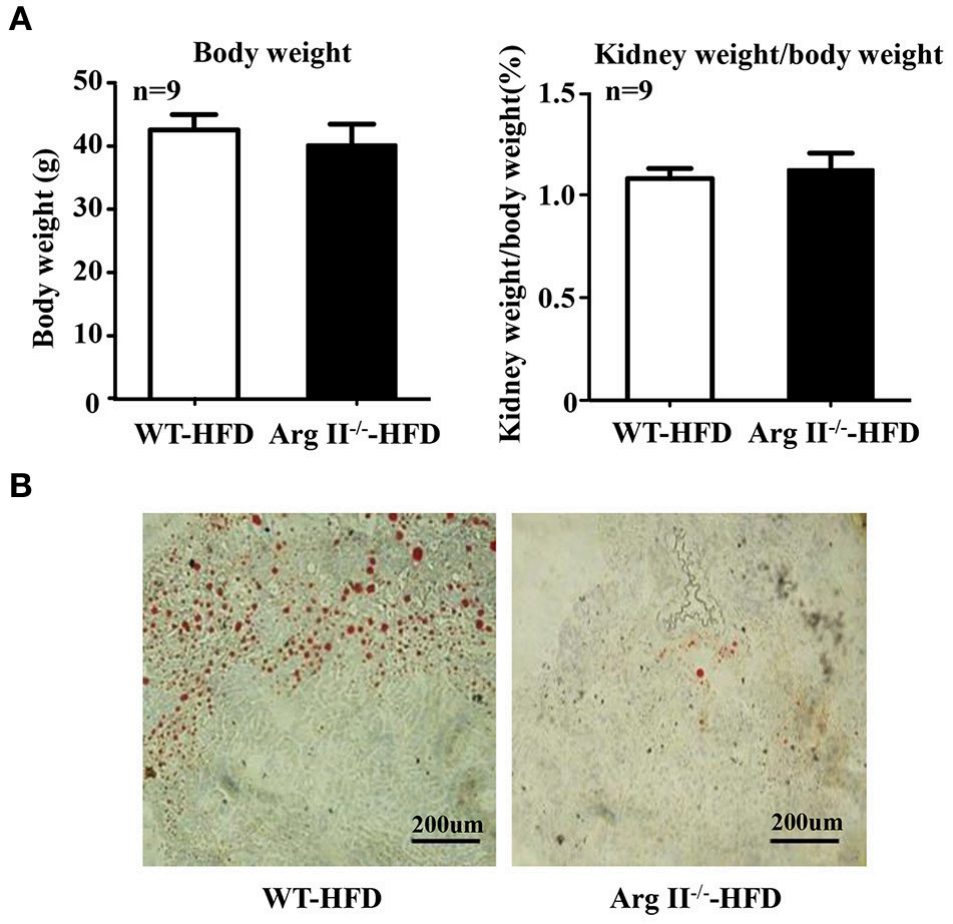
**Body weight, kidney weight/body weight ratio, and renal lipid accumulation in obesity**. Starting at the age of 7 weeks, WT and Arg-II^−/−^ mice were fed with HFD for 14 weeks. **(A)** Body weight and ratio of kidney weight/body weight after 14 weeks HFD; *n* = 11 for WT mice, *n* = 9 for Arg-II^−/−^ mice. **(B)** Representative images of Oil Red O staining of renal section. *n* = 9/group.

No difference in body weight and kidney weight/body weight ratio was observed between WT and Arg-II^−/−^ mice fed HFD (Figure 4A). Lipid accumulation in kidney was frequently observed in the obese WT mice, whereas this was not the case in the Arg-II^−/−^ mice (Figure 4B). The results indicate that Arg-II deficiency protects against ectopic renal lipid accumulation in diet-induced obesity.

### Arg-II-deficiency prevents renal oxidative oxygen species (ROS) generation, adhesion molecule expression and glomerulus mesangial expansion

**Figure 5:**
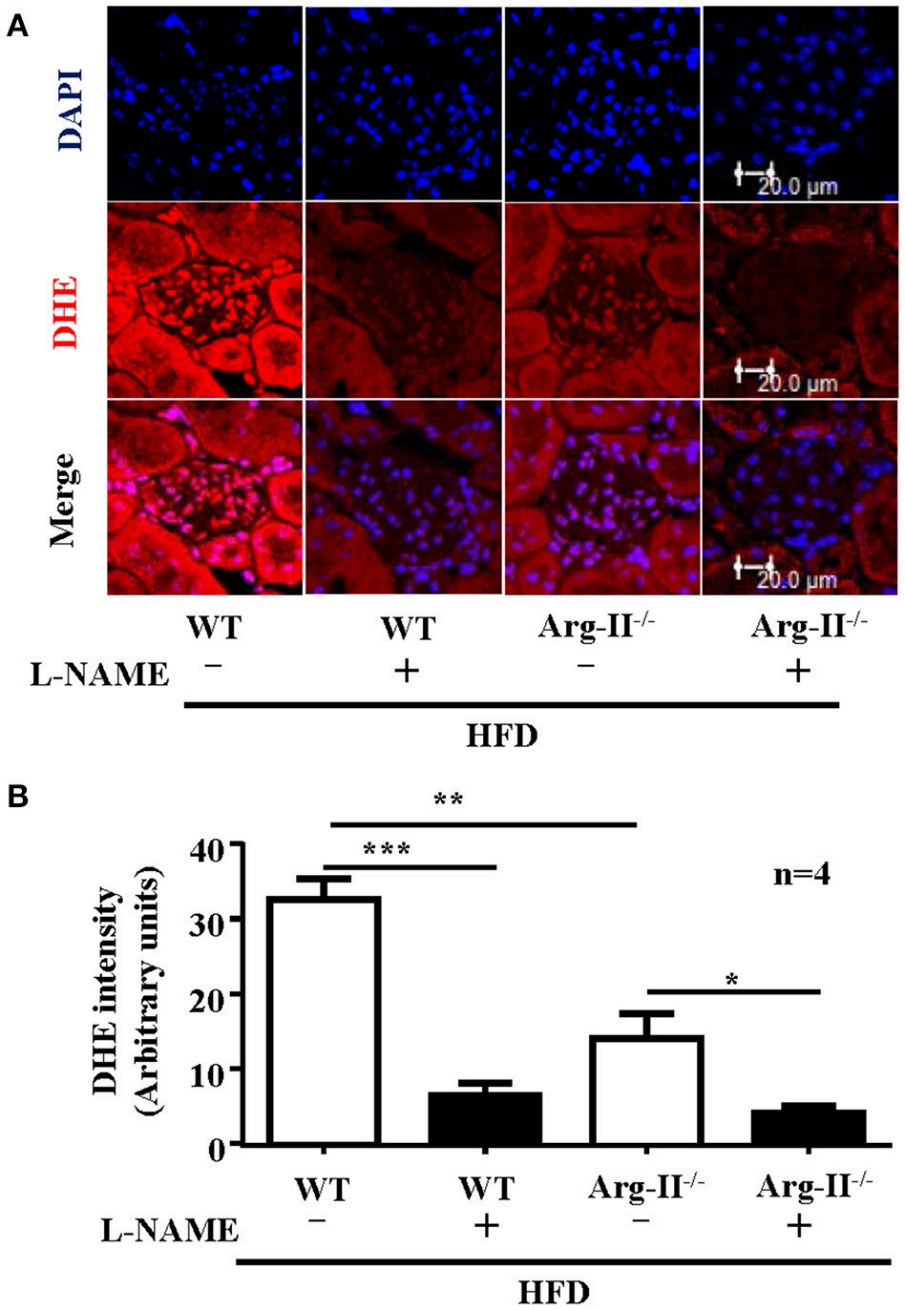
**Arg-II-deficiency decreases renal ROS production in obesity. (A)** Immuno-fluorescence confocal microscopy showing ${\text{O}}_{2}^{-}$ level in glomerulus detected by DHE staining (red). Kidney cryosections were pre-treated with or without NOS inhibitor L-NAME (1 mmol/L, 1 h) prior to the DHE staining. Nuclei were counter stained by DAPI (blue). **(B)** Bar graphs show quantification of DHE signals. *n* = 4, ^*^p < 0.05, ^**^p < 0.01, ^***^p < 0.001 between the indicated groups.

**Figure 6:**
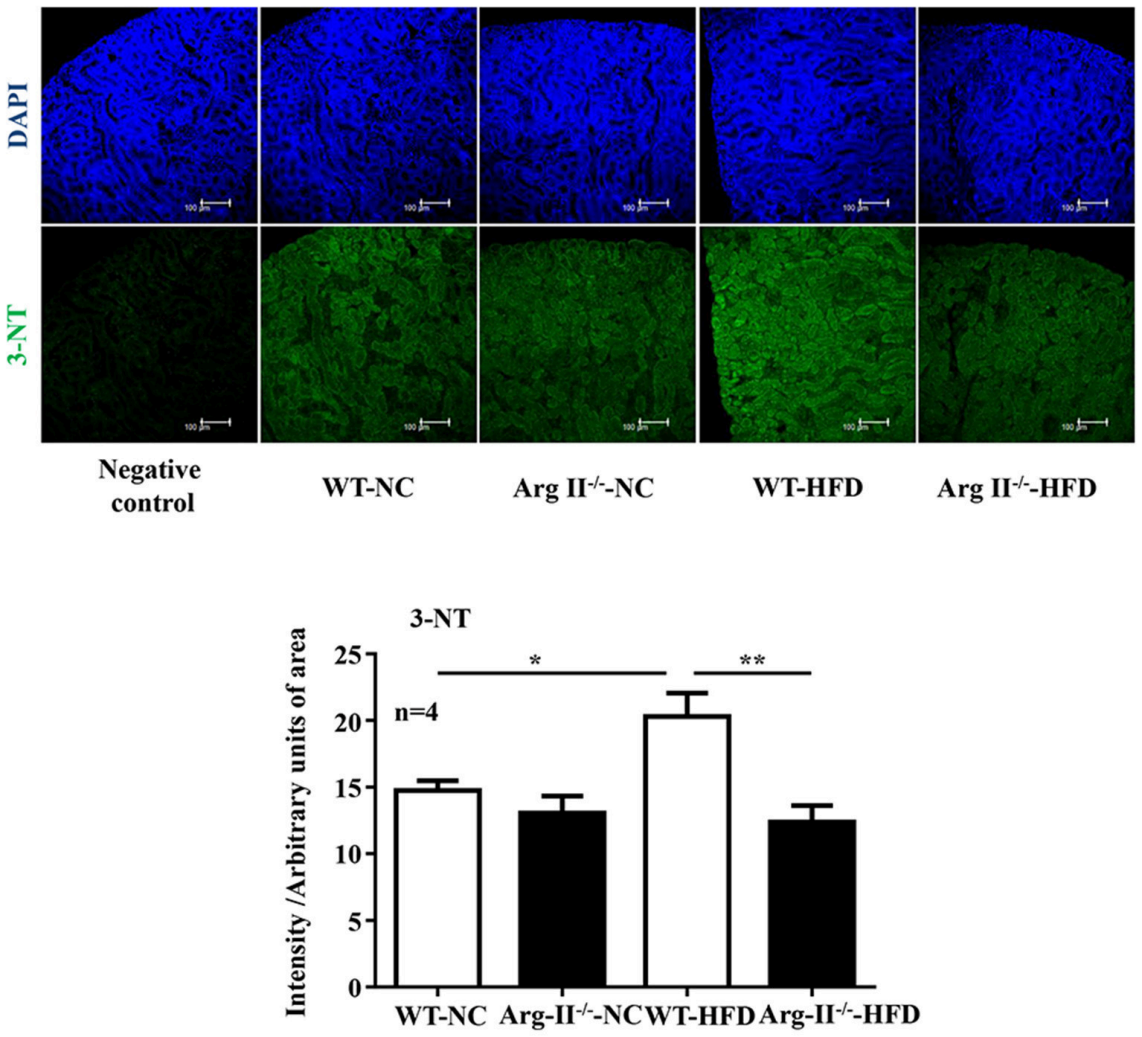
**Arg-II deficiency prevents obesity-associated increase in renal peroxynitrite formation**. Confocal immunofluorescence staining of 3-NT (green) and DAPI (blue) in the kidney of WT and Arg-II^−/−^ mice fed either normal chow (NC) or high fat diet (HFD). Representative images of individual staining and merged images are shown (*n* = 4). Quantifications of 3-NT signals are shown in the bar graphs. ^*^p < 0.05, ^**^p < 0.01.

**Figure 7:**
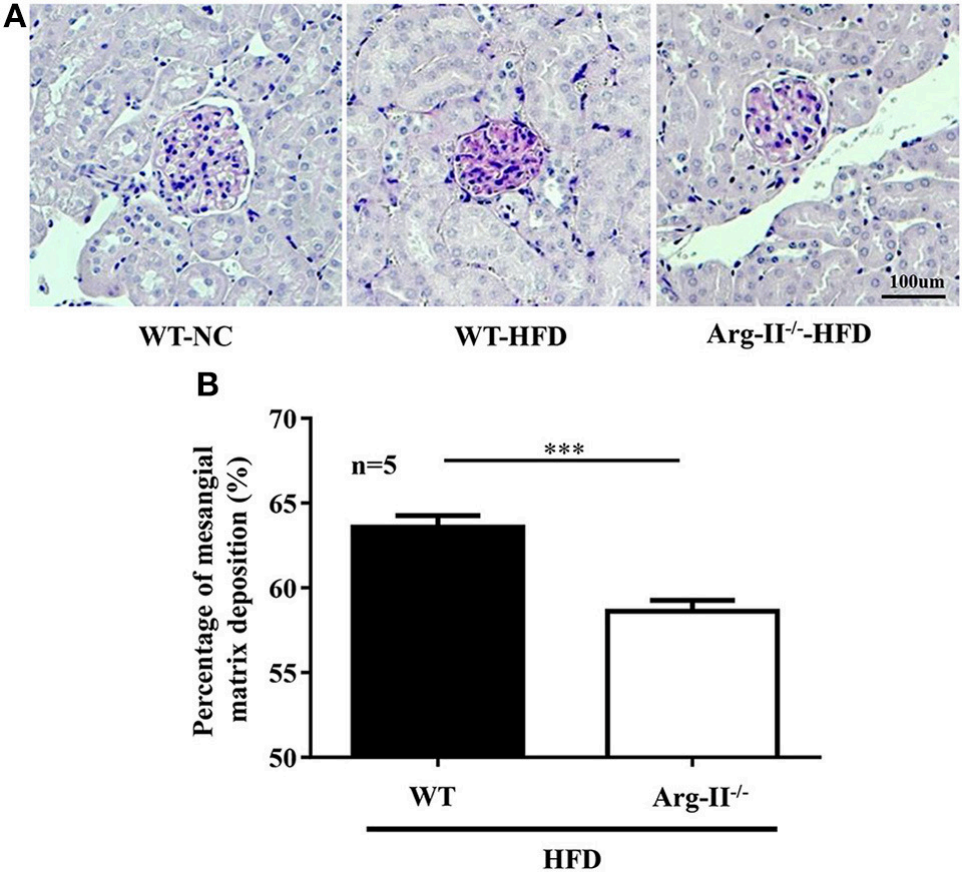
**Arg-II-deficiency protects against mesangial expansion in obesity. (A)** PAS staining (magenta) of paraffin-embedded kidney sections shows decreased mesangial expansion in Arg-II^−/−^ mice as compared to WT mice on HFD. Nuclei were counter stained by hematoxylin (blue). Kidney from a WT mice on NC diet was used as a control. **(B)** Bar graphs show quantification of mesangial expansion. *n* = 5, ^***^p < 0.001 vs. WT-HFD group.

**Figure 8:**
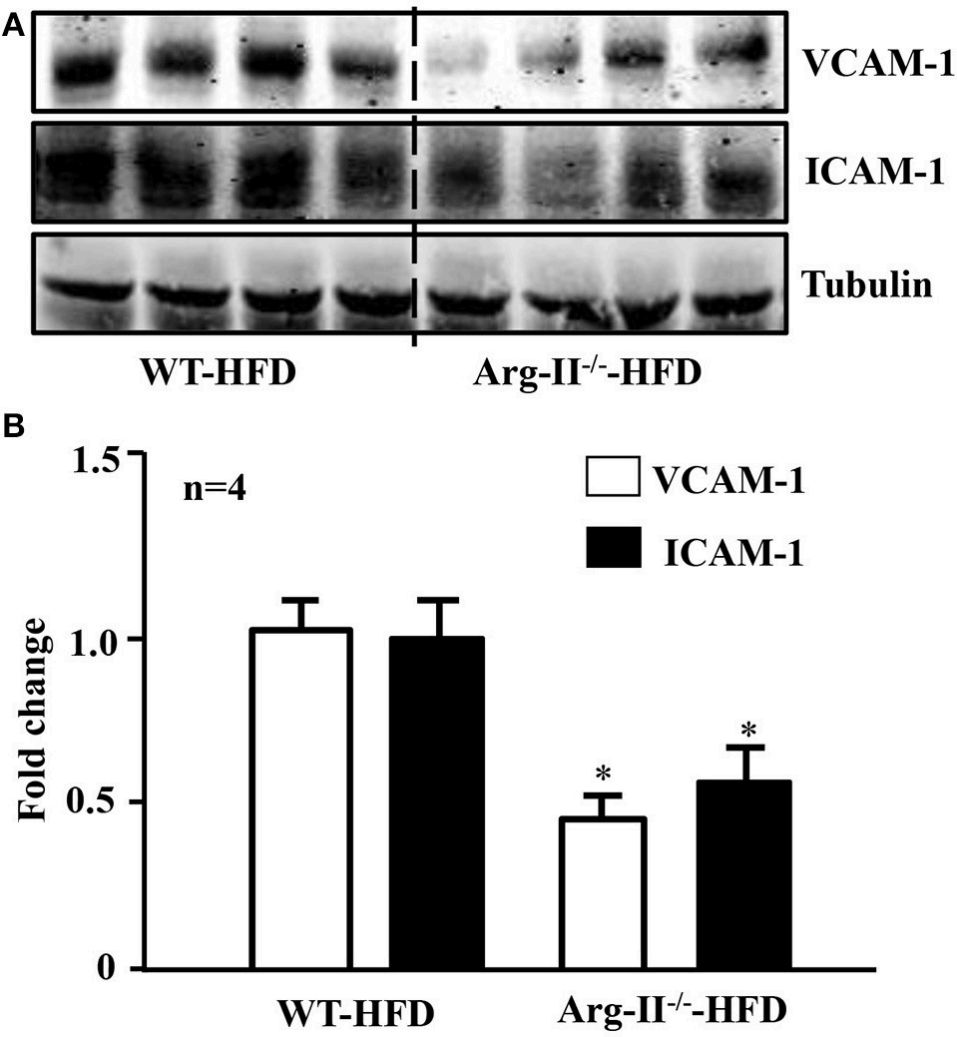
**Arg-II-deficiency reduces adhesion molecule levels in the kidney in obesity. (A)** Immunoblotting analysis of VCAM-1 and ICAM-I expression in the kidney of WT and Arg-II^−/−^ mice fed HFD. **(B)** Bar graphs show quantification of VCAM-1 and ICAM-I expression. *n* = 4, ^*^p < 0.05 between indicated groups.

In the WT mice fed HFD, there was a significant higher level of ROS generation (DHE staining) in the glomeruli as compared to the Arg-II^−/−^ mice on HFD (Figure 5). Interestingly, the ROS generation was inhibited by the NOS inhibitor L-NAME (10^−3^ mol/L, Figure 5), demonstrating NOS-uncoupling in obesity kidney. Similarly, peroxynitrite levels as demonstrated by 3-NT staining were increased in HFD fed WT mice and this obesity-induced increase in peroxynitrite was prevented in the Arg-II^−/−^ mice (Figure 6). Moreover, Arg-II^−/−^ mice revealed decreased mesangial expansion (Figure 7) and lower levels of renal ICAM-1 and VCAM-1 as demonstrated by immunoblotting (Figure 8).

## Discussion

In this study, we demonstrate that HFD-induced obesity is associated with renal injury markers, i.e., oxidative stress, as assessed by increased DHE staining and peroxynitrite levels, enhanced levels of inflammatory adhesion molecules, mesangial expansion, and more frequent lipid accumulation in kidney, which are characteristics of diabetic kidney disease as demonstrated by several studies in the same animal model (Lal et al., 2000; DeRubertis et al., 2004; Menini et al., 2006; Fioretto et al., 2008; Deji et al., 2009; Declèves et al., 2011; Cui et al., 2013). These renal alterations are important mechanisms leading to end stage renal disease (ESRD) associated with obesity. Importantly, our current study provides novel evidence demonstrating that diet-induced obesity enhances renal Arg-II level and that genetic ablation of Arg-II protects mice from the renal pathological changes in diet-induced obesity.

### Role of Arg-II in diabetic kidney disease

It is well known that kidney expresses abundant Arg-II (Morris et al., 2011). The physiological role of Arg-II in renal function is not known. Some studies demonstrate that Arg-II may participate in pathogenesis of renal diseases associated with type 1 diabetes in mouse models. For example, pharmacological inhibition of Arg-II in spontaneously diabetic Ins2^Akita^ mice or streptozotocin (STZ)-induced diabetic Dilute Brown Agouti (DBA) mice is able to attenuate renal injuries, including albuminuria and kidney macrophage infiltration (Morris et al., 2011; You et al., 2013). Also, mice with genetic Arg-II deficiency are protected from renal injuries in STZ-induced diabetes (Morris et al., 2011). These studies suggest an important role of Arg-II in type 1 diabetic renal disease. However, another group demonstrates opposite effects, i.e., Arg-II deficiency accelerates some of the STZ-induced renal damages in mice (Romero et al., 2013). The discrepancy between the two studies is not clear. In accordance with the study in type 1 diabetic mouse model in which renal arginase activity is increased (Morris et al., 2011), we also demonstrate in the current study an increase in Arg-II levels and arginase activity in kidney of the obesity-associated type 2 mouse model. Since Arg-I is not detectable in the kidney, the increase in arginase activity in kidney is solely attributable to Arg-II.

### Arg-II is mainly localized in the straight segment of proximal tubules

In line with the observation in kidney, we have previously shown that in the same diet-induced obesity mouse model, Arg-II is upregulated in other cells such as macrophages and vascular endothelial cells (Yu et al., 2014). The increase in Arg-II in macrophages under HFD feeding promotes pro-inflammatory responses, contributing to obesity-associated glucose intolerance, insulin resistance, and nonalcoholic fatty liver diseases (NAFLD) as well as atherogenesis (Ming et al., 2012; Liu et al., 2016). In kidney, we demonstrate in this study that Arg-II is mainly expressed in the S3 straight segment of the proximal tubules as demonstrated by expression in the B0AT3 positive cells. In the glomeruli, however, no Arg-II signal could be detected by immunofluorescence analysis. At this stage, the mechanisms of upregulation of Arg-II in kidney are not known. In the vascular cells, Arg-II upregulation involves activation of mTOR/S6K1 and p38mapk signaling pathways (Yepuri et al., 2012; Xiong et al., 2013, 2014; Wu et al., 2015). Whether these mechanisms are also involved in the obesity-associated renal injury remains to be investigated.

### Arg-II causes renal oxidative stress through NOS-uncoupling in obesity

It has been shown that Arg-II upregulation in blood vessels of obesity mice causes endothelial dysfunction and eNOS-uncoupling, leading to decrease in NO production and increase in superoxide radical anion levels (Yu et al., 2014). Importantly, eNOS-uncoupling has been reported to contribute to the pathogenesis of diabetic nephropathy (Prabhakar et al., 2007). In line with these findings, we demonstrate increased expression levels and activity of Arg-II in kidney of diet-induced obesity mice, which causes renal oxidative stress due to NOS-uncoupling, since the NOS inhibitor, L-NAME, reduces ROS levels as assessed by DHE staining. NO can react with superoxide radical anion to produce peroxynitrite which is able to cause nitration of tyrosine residues of proteins, i.e., nitrosative stress (Förstermann and Sessa, 2012). The modification of proteins at tyrosine residues often leads to functional decline of enzyme activity. It has been shown that the mitochondrial antioxidant enzyme manganese superoxide dismutase (MnSOD) is modified by the nitrosative stress and contains nitrotyrosine in angiotensin II-induced hypertensive rat kidney, which may facilitate the oxidative/nitrosative stress and renal injury (Xu et al., 2006). Various studies provide evidence for the important role of oxidative/nitrosative stress in renal injury and disease progression (Ratliff et al., 2016; Sharma, 2016). In support of this finding, the oxidative/nitrosative stress as assessed by increased levels of superoxide radical anion and 3-NT in kidney of obesity mice is prevented in Arg-II^−/−^ animals despite no changes in body weight and kidney weight/body weight ratio. It is to mention that myeloperoxidase and other peroxidases are also involved in nitrotyrosine formation (Gaut et al., 2002). Functional roles of these enzymes in renal nitrosative stress in the mouse model could not be excluded and require further investigation.

Oxidative stress derived from eNOS-uncoupling caused by Arg-II is causatively involved in adhesion molecule expression in the endothelial cells (Yepuri et al., 2012). In line with this observation, our present study demonstrates that Arg-II and adhesion molecule expression in kidney are upregulated in obesity and that Arg-II deficiency reduces oxidative stress, decreases VCAM-1 and ICAM-1 levels in kidney of the obesity mouse model. Together, these results suggest that Arg-II indeed plays an important role in obesity-associated renal damage involving NOS-uncoupling. Since kidney expresses eNOS, iNOS, and nNOS (Prabhakar et al., 2007) and decreased renal NO bioavailability is linked to diabetic renal disease (Erdely et al., 2004; Prabhakar, 2005), the question whether each of these isoenzymes are uncoupled in kidney of obesity mice remains to be investigated. Another important question which remains to be investigated is how the proximal tubule straight segments interact with glomerulus cells and participate in diabetic renal injury.

### Role of Arg-II in renal lipid accumulation in obesity

It is important to point out that the Arg-II^−/−^ mice are protected from whole body glucose intolerance with improved insulin sensitivity and less NAFLD on HFD (Liu et al., 2016). Similar to the liver, we also found a decreased renal lipid accumulation in kidney as shown in our current study. The renal lipid accumulation in type 2 diabetes is well described in humans and animal models, which is considered as an important contributor to progression of diabetic nephropathy in obesity (Jiang et al., 2005). The underlying mechanisms are attributable to the increased expression of the transcriptional factors SREBP-1c, the master regulator of lipogenesis and suppressed renal lipolysis (Jiang et al., 2005; Kume et al., 2007). The results of our current study suggest an important role of Arg-II in obesity-associated renal diseases. It remains to be investigated how important the renal Arg-II is in the development of obesity-associated kidney damage. For this purpose, renal specific Arg-II knockout mouse model shall be generated in the future.

## Conclusions

We demonstrate that Arg-II deficiency in mouse protects against obesity-associated renal alterations. The results suggest that targeting Arg-II may represent a promising therapeutic strategy for diabetic nephropathy.

## Author contributions

JH, AR, and YX: acquisition, analysis, or interpretation of data for the work; preparation of figures; critically revised the manuscript for important intellectual content. XM and ZY: design of the work; analyzed and interpreted primary research papers, drafted the manuscript and critically revised the manuscript for important intellectual content. FV: generated and validated antibody against B0AT3 and critically revised the manuscript for important intellectual content. JM: interpreted primary research papers, critically revised the manuscript for important intellectual content. All authors agree to be accountable for the content of the work.

## Funding

This work was supported by the Swiss National Science Foundation (31003A_159582/1), Swiss Heart Foundation, and National Centre of Competence in Research Program (NCCR-Kidney.CH).

### Conflict of interest statement

The authors declare that the research was conducted in the absence of any commercial or financial relationships that could be construed as a potential conflict of interest.
